# Use of flow cytometry for the adhesion analysis of *Streptococcus pyogenes *mutant strains to epithelial cells: investigation of the possible role of surface pullulanase and cysteine protease, and the transcriptional regulator Rgg

**DOI:** 10.1186/1471-2180-6-18

**Published:** 2006-02-24

**Authors:** Jukka Hytönen, Sauli Haataja, Jukka Finne

**Affiliations:** 1 Department of Medical Biochemistry and Molecular Biology, University of Turku, Kiinamyllynkatu 10, FI-20520 Turku, Finland

## Abstract

**Background:**

Flow cytometry based adherence assay is a potentially powerful but little used method in the study of bacterial binding to host structures. We have previously characterized a glycoprotein-binding activity in *Streptococcus pyogenes *called 'strepadhesin' binding to thyroglobulin, submaxillar mucin, fetuin and asialofetuin. We have identified surface-associated pullulanase (PulA) and cysteine protease (SpeB) as carriers of strepadhesin activity. In the present paper, we investigated the use of flow cytometry as a method to study the binding of Rgg, SpeB and PulA knock-out strains to cultured human epithelial cells.

**Results:**

Streptococcal mutants were readily labelled with CFDA-SE and their binding to epithelial cells could be effectively studied by flow cytometry. A strain deficient in Rgg expression showed increased binding to the analyzed epithelial cell lines of various origin. Inactivation of SpeB had no effect on the adhesion, while PulA knock-out strains displayed decreased binding to the cell lines.

**Conclusion:**

These results suggest that the flow cytometric assay is a valuable tool in the analysis of *S. pyogenes *adherence to host cells. It appears to be an efficient and sensitive tool for the characterization of interactions between the bacteria and the host at the molecular level. The results also suggest a role for Rgg regulated surface molecules, like PulA, in the adhesion of *S. pyogenes *to host cells.

## Background

Flow cytometry has been established as a standard tool in the characterization of eukaryote cells and their interactions. Flow cytometric adherence assay has in recent years also been adopted for the analysis of interactions between bacteria and eukaryotic cells [1,2]. The results suggest some benefits over conventional assays but the method has not yet been much used. In conventional adherence assays, bacteria are allowed to attach to solid-phase immobilized cells, and after washings, released by lysing the host cells and enumerated either by time-consuming plate cultures or by counting the bacteria using a microscope. In contrast, the flow cytometric assay displays characteristics such as fast performance, large through-put of analyzed cells, and the avoidance of washing steps. This should allow even the detection of weak interactions between bacteria and host cells. In addition, since the bacteria and host cells interact in liquid phase, other types of bacterium-cell interactions may take place as compared to solid-phase assays. On the other hand, mechanical detachment of the cell for flow cytometry based adhesion assay may in theory create artificial binding activities, which are avoided in conventional monolayer adhesion assays.

*Streptococcus pyogenes *(GAS) is the etiologic agent of a variety of human diseases, like pharyngitis and erysipelas. It causes substantial morbidity in industrialized countries, and is a leading cause of rheumatic heart disease especially in the developing world [3]. Also, life-threatening infections, like necrotizing fasciitis and toxic shock-like syndrome have been on the increase since the beginning of the last decade [4,5]. An increasing number of *S. pyogenes *strains resistant to certain antibiotics have been reported world wide [6,7]. To develop novel therapeutic and preventive approaches against these infections, a thorough understanding of the interaction between *S. pyogenes *and the host is needed.

The adherence of pathogenic bacteria to a host is in many cases mediated by an interaction between adhesins on the surface of the microbe and receptors molecules, in many cases carbohydrates, on the host tissues. *S. pyogenes *expresses a large array of molecules on its surface implicated in adhesion to host structures. These are among others lipoteichoic acid [8], M protein [9,10], various fibronectin-binding proteins like protein F (SfbI) [11,12], protein F2 [13], fibronectin-binding protein FBP54 [14], serum opacity factor/SfbII [15,16], 28 kDa fibronectin-binding protein [17], Fba [18], PFBP [19] and SfbX [20], and the hyaluronic acid capsule [21,22]. Although several interactions between *S. pyogenes *and structures of the host have been characterized, the issue of GAS adhesion remains open. This is clearly attributed to the fact that GAS expresses a wide variety of adhesins which recognize many host structures leaving the role of specific molecules incompletely understood.

We wanted to test the applicability of the flow cytometry based cell adhesion assay in the analysis of the binding of *S. pyogenes *surface protein mutant strains to human epithelial cell lines. We have previously characterized 'strepadhesin' activity as a novel mechanism mediating the binding of *S. pyogenes *to glycoproteins thyroglobulin, submaxillar mucin, fetuin and asialofetuin [23,24]. We have shown that the activity is present in the majority of strains studied, and that it is upregulated by Mga and downregulated by Rgg, both of which are central transcriptional regulators of *S. pyogenes *gene expression. We have also identified two molecules that carry strepadhesin activity on streptococcal surface, streptococcal cysteine protease, SpeB, and streptococcal pullulanase, PulA [24,25]. SpeB is a 28 kDa extracellular protease and surface associated molecule produced by most GAS strains. PulA is a 129 kDa surface adhesin of GAS with pullulan and starch hydrolysing activities. In the present study, we analyzed the binding of SpeB and PulA surface protein mutants, as well as an Rgg-regulator deficient mutant strain to an array of human epithelial cell lines with flow cytometry.

## Results and discussion

### Flow cytometry based adherence assay

**Figure 1:**
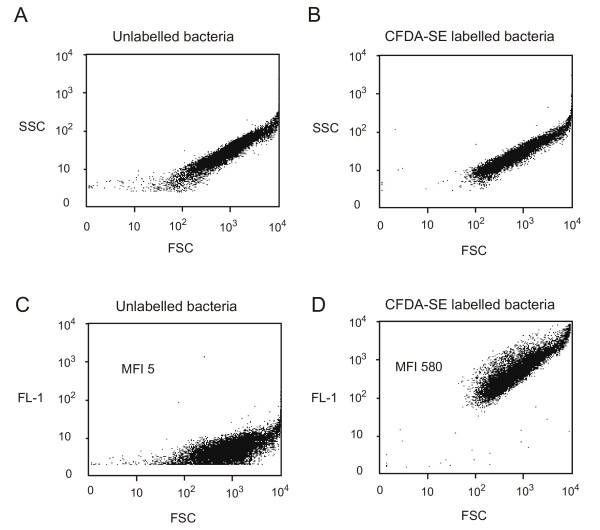
Labelling of the NZ131*rgg *bacteria with CFDA-SE. Bacteria were grown overnight, washed, labelled with CFDA-SE for 20 min at 37°C, and analyzed with a flow cytometer. (A) Distribution of unlabelled bacteria and (B) of labelled bacteria in the dot plot display mode of forward scatter (FCS) versus side scatter (SSC). The efficiency of the labelling was analyzed by measuring the fluorescence of the bacteria on channel FL1. The labelling of the bacteria is seen as a shift of the bacteria along the FL1 axis in the scatter plot (D). The background fluorescence of the unlabelled bacteria is shown in panel (C) (MFI, mean fluorescence intensity).

The method used for the study of the adhesion of the streptococcal mutants to epithelial cells was adapted by modification from previously described reports [1,2]. The bacteria were labelled with CFDA-SE, a non-fluorescent membrane permeable ester, which is converted to a fluorescent molecule by non-specific intracellular esterases, and covalently linked to intracellular proteins through its succinimidyl group [26]. Streptococci were found to be effectively labelled with CFDA-SE as shown in Fig. 1. For example, there is a ~100 fold increase in the fluorescence of labelled NZ131*rgg *bacteria compared to unlabelled bacteria (Fig. 1C and 1D).

The SCC cell lines used in the adhesion experiments were derived from epithelial squamous cell carcinomas originating from the human oral cavity (SCC1A: gingiva, SCC8: larynx, SCC24A: tongue and SCC60A: tonsil) and from nasal skin (SCC12A). We chose to use these cell lines because they originate from the two natural habitats of group A streptococcus and they are easy to culture. Also, Sethman and others [2] reported that buccal epithelial cells, which are often used as a substrate in studies with oral streptococci, are not ideal for *S. pyogenes *adhesion assays due to non-specific binding. However, we are aware of the fact that malignant cells do not necessarily express the same sets of surface proteins and carbohydrates as native cells, and thus the results that we obtained using these cells lines must be interpreted with caution.

**Figure 2:**
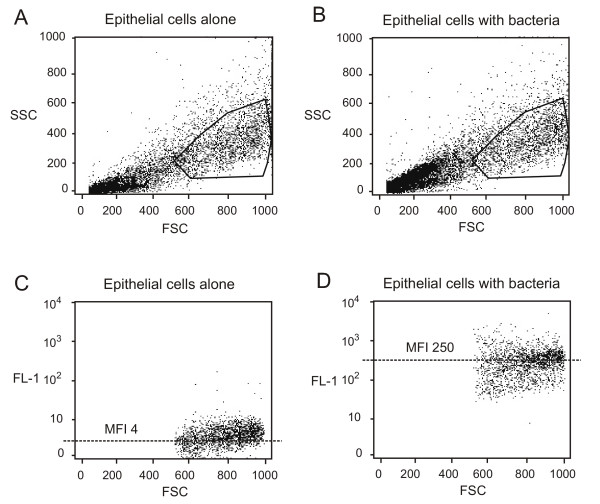
Flow cytometric analysis of human epithelial cells alone and after incubation with labelled streptococci. (A) Initial analysis of the cells (SCC24A) in the dot plot display mode of forward scatter (FCS) versus side scatter (SSC). The core population of the cells is surrounded by a gate, and cellular debris is seen in the lower left hand corner. (B) Dot plot display mode of FCS versus SSC after the incubation of the cells with labelled bacteria (NZ131*rgg*). (C) Autofluorescence of the gated cells in dot plot display mode of FL1 versus FCS. (D) Fluorescence of the gated cells after incubation of the cells with labelled bacteria. (MFI, mean fluorescence intensity).

The initial analysis of the epithelial cells revealed a population of cells with some cellular debris (Fig. 2A). Also, bacterial aggregates were occasionally detected within the size range of the epithelial cells (Fig. 2B), but were discarded by running a sample of bacteria alone and excluding this data from the analysis of epithelial cell fluorescence. Thus, the flow cytometer was set to include a subpopulation of epithelial cells and to gate out cellular debris and bacterial clumps (Fig. 2A and 2B). The epithelial origin of the gated cells was confirmed with a FITC-labelled Ber Ep4 antibody specific for epithelial cells (data not shown). Epithelial cells are weakly autofluorescent which is seen in the scatter plot (Fig. 2C). However, epithelial cells incubated with fluorescent bacteria for 1 h at RT shifted along the fluorescence axis in the scatter plot indicating attachment of bacteria to the cells (Fig. 2D). There is a ~60 fold increase in the MFI of cells incubated with bacteria compared to the epithelial cells alone. The incubation of the cells with unlabelled bacteria in the absence of labelled bacteria did not affect the fluorescence of the cells (data not shown). The results indicate that streptococci are efficiently labelled with CFDA-SE, and that the binding of streptococci to epithelial cells can be assayed using this flow cytometric approach.

### Adhesion of Rgg deficient streptococci to human epithelial cells

**Figure 3:**
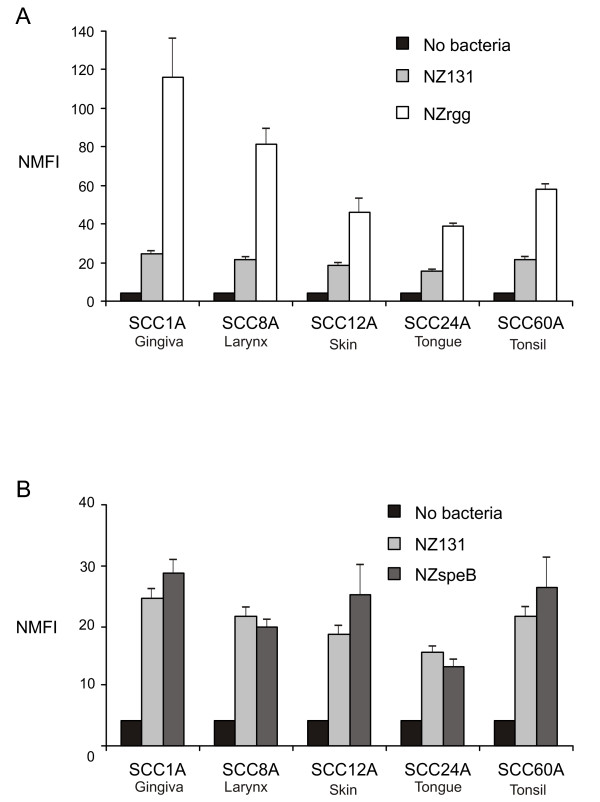
The adhesion of NZ131*rgg *(A) and NZ131*speB *(B) streptococci to SCC1A, SCC8, SCC12A, SCC24A and SCC60A cell lines as compared to the NZ131 wild type strain. The normalized mean fluorescence intensities (NMFI) of the cells after incubation with the bacteria are shown as columns with standard errors.

The role of Rgg regulated molecules in the adhesion of streptococci to cells originating from various tissues of the human oral cavity (SCC1A, SCC8, SCC24A and SCC60A) and from the skin (SCC12A) was next investigated. Rgg has been shown to be a central regulator of virulence factor expression in GAS [27], and it is also involved in the regulation of the strepadhesin activity [25]. Rgg appears to be a negative regulator of the transcription of at least part of the genes it regulates, since in the Rgg deficient bacteria the expression of several virulence-associated genes is upregulated [28]. Fluorescently labelled NZ131 wild-type and NZ131*rgg *[29] mutant bacteria were allowed to adhere to the cells for 1 h at RT and the fluorescence of the cells was measured (Fig. 3A). The binding of NZ131*rgg *bacteria to all the studied cell lines was increased two to four fold as compared to the wild-type strain. These results demonstrate that streptococcal adherence to human epithelial cells is upregulated in the absence of the Rgg regulator, and thus, in the presence of several surface molecules that are missing in the wild type strain. Also, the results demonstrate that there is no major difference in NZ131 adhesion either between the SCC cell lines originating from the different parts of the oral cavity, or between the oral cell lines and the cell line derived from the skin. NZ131*rgg *adheres most avidly to the cells originating from gingiva.

### Adhesion of SpeB deficient streptococci to human epithelial cells

The role of SpeB, a secreted and surface associated virulence factor in the adhesion of streptococci to SCC1A, SCC8, SCC24A, SCC60A and SCC12A cells was next investigated. Fluorescently labelled NZ131 wild-type and NZ131*speB *[30] mutant bacteria were allowed to adhere to the cells for 1 h at RT and the fluorescence of the cells was measured (Fig. 3B). Adhesion NZ*speB *bacteria to epithelial cells was not much changed as compared to the wild type strain. However, there was a slight, although not statistically significant, trend towards increased adherence of SpeB-deficient bacteria to SCC1A, SCC12A and SCC60A cells. The increase may be related to the fact that SpeB is an enzyme known to cleave proteins from streptococcal surface [31]. Therefore, the absence of SpeB production may result in decreased shedding of GAS surface proteins, like the M-protein, which may in turn lead to increased adhesion.

### Contribution of pullulanase to the cell adhesion

**Figure 4:**
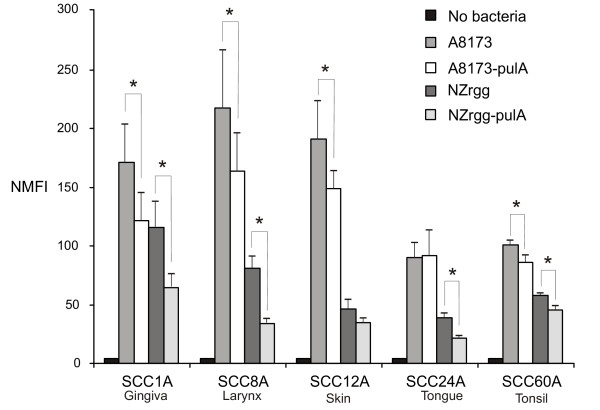
The role of streptococcal pullulanase in the adherence of streptococci to human epithelial SCC1A, SCC8, SCC12A, SCC24A and SCC60A cell lines. The normalized mean fluorescence intensities (NMFI) of cells after incubation with A8173, A8173*pulA*, NZ131*rgg *and NZ131*rgg-pulA *bacteria are shown as columns with standard errors. Statistically significant differences between PulA deficient and parental strains are indicated with asterisks (* p < 0.05).

We have previously shown that the expression of streptococcal pullulanase is increased in Rgg deficient NZ131*rgg *strain, and that pullulanase carries strepadhesin glycoprotein-binding activity [25]. In order to analyse the contribution of pullulanase to epithelial adherence, the binding of pullulanase-deficient NZ131*rgg-pulA *bacteria to epithelial cells was analyzed. Also, another pullulanase-deficient derivative was generated as previously described from A8173 wild type strain, which has high strepadhesin activity. Parental NZ131*rgg *and A8173 strains and mutant NZ131*rgg*-*pulA *and A8173*pulA *bacteria were allowed to adhere to the cells for 1 h at RT. Statistical analysis showed that the difference between A8173 and A8173*pulA *in adherence to SCC1A, SCC8, SCC12A and SCC60A, and between NZ131*rgg *and NZ131*rgg*-*pulA *in adherence to SCC1A, SCC8, SCC24A and SCC60A was significant (p < 0.05) (Fig. 4). These results suggest that streptococcal pullulanase may have a role in the adherence of GAS to epithelial cells.

## Conclusion

Use of flow cytometry has been adopted during the recent years for the analysis bacterial binding to host cells. The flow cytometric assay is faster than the conventional assays and the number of analyzed cells is bigger. In addition, the flow cytometry based assay is presumed to allow the detection of weak and/or additional bacterium-cell interactions as compared to the conventional solid phase adhesion assays. This may, in fact, be the case with the streptococcal strains of this study since our conventional adherence experiments with the wild type bacteria and PulA deficient mutants demonstrated no difference in the binding to SCC cells (J. Hytönen, unpublished observation), while with the flow cytometric assay there is a statistically significant difference between the strains in adhesion to majority of the analyzed cell lines. In summary, the results of the present study suggest that the flow cytometric assay of streptococcal adhesion to human epithelial cells is a valuable tool in the characterization of the interactions between *S. pyogenes *and the host.

## Methods

### Bacterial strains and culture conditions

*Streptococcus pyogenes *NZ131 wild-type (type M49) and the *speB*- and *rgg*-mutant strains were kind gifts from M. Chaussee, National Institute of Health, Hamilton, Montana, USA [29,30]. *S. pyogenes *NZ131*rgg-pulA *double mutant strain has been previously characterized [25]. *S. pyogenes *clinical isolate A8173 (type M2) was provided by K. Kunnas, National Public Health Institute, Kuopio, Finland, and the pullulanase deficient derivative A8173*pulA *was constructed during this study. Streptococcal strains were grown on Todd-Hewitt (Difco) plates or media (THY) supplemented with 0.5 % yeast extract (Biokar Diagnostics). All bacteria were stored at -70°C in growth medium containing 15 % glycerol. When appropriate, antibiotics were added to the culture media to the following concentrations: erythromycin 3 μg/ml, kanamycin 500 μg/ml.

### Preparation of epithelial cells

Squamous cell carcinoma cell lines SCC1A (gingiva), SCC8 (larynx), SCC24A (tongue) and SCC60A (tonsil) originating from various tissues of human oral cavity and from the skin SCC12A (nasal skin) were obtained from R. Grénman, Department of Otorhinolaryngology, University of Turku, Finland. The cells were cultured in Dulbecco's Modified Eagle's Medium supplemented with 5% foetal bovine serum supplemented with 10 μg/ml gentamicin sulphate and 100 μg/ml streptomycin sulphate. The cells were cultured to near-confluence, washed once in PBS, detached mechanically without any proteases and suspended in cold PBS.

### Preparation of fluorogenic label

Carboxyfluorescein diacetate succinimidyl ester (CFDA-SE, Molecular Probes) was dissolved in dimethylsulphoxide at a concentration of 10 mg/ml, and was further diluted in ethanol to give a stock solution concentration of 1.7 mg/ml. The stock solution was stored at -20°C. For use, the stock solution was diluted in PBS to a concentration of 12 μg/ml.

### Labelling of the bacteria

CFDA-SE staining of streptococci was performed based on a previously described method [1]. Bacteria grown overnight in THY were washed in PBS and re-suspended in 2 ml PBS to a concentration of ca. 10^10 ^cfu/ml. Two millilitres CFDA-SE in PBS was added to the bacterial suspension in 15 ml centrifuge tubes and the cells were incubated with end-over-end rotation at 37°C for 20 min. The bacteria were harvested by centrifugation at 3000 × g at 4°C, washed twice with 12 ml of ice-cold PBS, and re-suspended to a concentration of 10^9 ^cfu/ml. The majority of bacteria remained viable following CFDA-SE staining as revealed by plate counts (not shown).

### Adherence assay

Labelled bacteria were added to 200 μl of the epithelial cell suspension to yield a final concentration of 10^8 ^cfu/ml of the bacteria and 10^5^/ml the cells. The tubes were incubated with gentle agitation for 1 h at RT. After the adhesion step, the cells were fixed with no washings by adding 1 vol. of 4% w/v paraformaldehyde in PBS. The tubes were gently inverted and kept on ice until the samples were analyzed using the flow cytometer.

### Flow cytometry

The fluorescent intensity of the epithelial cells with adherent streptococci was measured with FACSCalibur equipment (Becton Dickinson). The data obtained from the flow cytometer were analyzed using CELLQuest software (Becton Dickinson). The initial analysis of the cells was carried out in the dot plot display mode of forward scatter (FCS) versus side scatter (SSC). Each point represents an individual epithelial cell. FCS in the *x*-axis is a measure of the size of the detected particle and SSC in the *y*-axis measures the cellular granularity. Based on the FCS v. SSC analysis of the cells and staining of the cells with the FITC-conjugated Ber Ep4 antibody (DAKO) specific to epithelial cells, the flow cytometer was set to include epithelial cells and to gate out cellular debris and non-adherent bacteria. The fluorescence of the cells was detected at channel FL1, and is shown as scatter plots of FCS in the *x*-axis v. FL1 in the *y*-axis. The numerical results are expressed either as geometric mean fluorescent intensity (MFI) values of a cell population (Fig. 1C and 1D; Fig. 2C and 2D) or as geometric MFI values of a cell population normalized based on the mean fluorescent intensities of the labelled bacteria (NMFI = MFI of epithelial cell with adherent streptococci/MFI of streptococci; Fig. 3 and 4). The cell-bacteria incubations were carried out in three parallel tubes and the experiments were repeated two or three times with new batches of epithelial cells and labelled bacteria. Statistical analyses were carried out using SAS System software version 9.1.3 Analyses of binding were done using general linear mixed models. Cell-type and bacterial strain were used as fixed effects and repetition as repeated factor. Same cell material was taken account as random effect. Because there was significant interaction with cell-type and bacteria the differences between bacteria were calculated within each cell-type and were adjusted with Bonferroni-method. P-values less than 0.05 were considered statistically significant.

### Mutagenesis of *pulA *in A8173

The mutagenesis of *pulA *in A8173 was performed as described previously [25]. Briefly, an internal fragment of the *pulA *gene (nucleotides 297–476) was cloned into a suicide vector pSF151 [32]. The plasmid was propagated in *E. coli *K12 GM2163 (New England Biolabs) and 10 μg of the purified plasmid was electroporated into competent A8173 bacteria as described [33]. Recombinants were selected on THY plates containing 500 μg/ml of kanamycin. The integration of the suicide plasmid in *pulA *gene was confirmed by PCR with primers specific to pSF151 (5'-TTAGCTCACTCATTAGGCAC-3') and to 5' upstream region of *pulA *gene (5'-GGACACGGTATTAGAGACCAAG-3').

## List of abbreviations

GAS: group A streptococcus

PulA: streptococcal pullulanase

SpeB: streptococcal cysteine protease

Mga: multiple gene activator of GAS

Rgg: regulator gene of GTF; first described in *Streptococcus gordonii*

SCC: squamous cell carcinoma

MFI: mean fluorescence intensity

NMFI: normalized mean fluorescence intensity

## Authors' contributions

JH had the primary responsibility of the planning of the study and carried out the experiments, SH contributed to the planning of the study and JF directed the design and execution of the project. All authors have read and approved the manuscript.
